# Bioaccessibility of Flavones, Flavanones, and Flavonols from Vegetable Foods and Beverages

**DOI:** 10.3390/biology13121081

**Published:** 2024-12-22

**Authors:** Alice Cattivelli, Melissa Zannini, Maddalena De Angeli, Domenico D’Arca, Vincenzo Minischetti, Angela Conte, Davide Tagliazucchi

**Affiliations:** 1 Nutritional Biochemistry Lab, Department of Life Sciences, University of Modena and Reggio Emilia, Via Amendola 2, 42122 Reggio Emilia, Italy; alice.cattivelli@unimore.it (A.C.); melissa.zannini@unimore.it (M.Z.); maddalena.deangeli@unimore.it (M.D.A.); angela.conte@unimore.it (A.C.); 2 Department of Biomedical, Metabolic and Neural Sciences, University of Modena and Reggio Emilia, 41121 Modena, Italy; domenico.darca@unimore.it (D.D.); vincenzo.minischetti@unimore.it (V.M.)

**Keywords:** phenolic compounds, mass spectrometry, food matrix, gastro-intestinal digestion, bioavailability

## Abstract

The bioaccessibility of flavonoids is of paramount importance in determining their bioavailability and biological effects. Bioaccessibility is influenced by several aspects, comprising the food matrix and the structure of flavonoids. In the present study, the bioaccessibility of different classes of flavonoids (flavanones, flavones, and flavonols) was investigated after in vitro gastro-intestinal digestion of beverages and vegetables. O-glycosylated flavanones were stable during in vitro digestion and easily released from the food matrix. Otherwise, C-glycosylated flavanones displayed a lower bioaccessibility index. Similarly, flavones exhibited a high bioaccessibility index in beverages and vegetables, with the O-glycosylated forms being more stable than the C-glycosylated. Flavonols displayed different stability under gastro-intestinal conditions depending on their structure. The presence of a catechol moiety in the B-ring, as observed in 3-O-glycosylated quercetins, negatively impacted flavonol stability in comparison with kaempferol derivatives that lack the catechol moiety. Indeed, the presence of more than one sugar or the glycosylation of the OH group in the B-ring improved the digestive stability of quercetin derivatives. For flavonols, a clear food matrix effect was observed by comparing the bioaccessibility in beverages and vegetable foods. These findings may aid in improving the comprehension of the biological effects of flavonoids and flavonoid-rich foods.

## Simple Summary

Flavonoids are secondary plant metabolites that exert some beneficial effects on the human body, being implicated in preventing some chronic diseases, such as cardio-vascular diseases, diabetes, and cancers. To fulfil their biological activity, flavonoids should be liberated from the food matrix throughout gastro-intestinal digestion, stable in the gastro-intestinal environment, and absorbed at the intestinal level or exercise their effects in the gastro-intestinal tract. Therefore, the study of the bioaccessibility on flavonoids (i.e., the release from the food matrix and the stability under gastro-intestinal conditions) is of paramount importance to understand which molecules are truly implied in the healthful effects of these compounds. In this work, the bioaccessibility of three different classes of flavonoids (flavanones, flavones, and flavonols) from selected beverages and vegetables was studied using an in vitro model that mimics gastro-intestinal digestion. The different classes of flavonoids were characterized by different behaviour during in vitro digestion. Flavones were the more stable compounds with a bioaccessibility index near 100%. Also, flavanones, especially the O-glycosylated derivatives, were quite stable during digestion. In general, bioaccessibility depends on the flavonoid structure and the food matrix. This study may aid in a better understanding of the beneficial effects of flavonoids.

## 1. Introduction

Numerous observational and clinical studies suggested strong scientific evidence of the health benefits of vegetable food consumption, as recently reviewed by Wallace et al. [1]. Most of the health effects related to vegetable food consumption have been associated with the presence of phenolic compounds [2].

Phenolic compounds are secondary metabolites present in plant and vegetable foods that have gathered great attention in the latest decades due to their supposed health benefits [3]. Numerous studies indicated that phenolic compounds and their related metabolites possessed in vitro antioxidant, anti-proliferative, neuroprotective, anti-inflammatory, and anti-diabetic activities [4,5,6,7,8].

In addition, phenolic compounds were found to be able to modulate gut microbiota by promoting a eubiosis condition [9,10]. Moreover, epidemiological and pre-clinical studies associated the consumption of phenolic compounds-rich foods with a lower incidence of cardio-vascular diseases, hypertension, some types of cancer, and diabetic complications [11].

Phenolic compounds may exert their health benefits directly in the gastro-intestinal tract where they can be present even at millimolar concentrations [12]. For example, phenolic compounds may inhibit in the gastro-intestinal tract some key metabolic enzymes involved in carbohydrate metabolism (such as α-amylase, α-glucosidase, and dipeptidyl-peptidase-IV), decreasing starch hydrolysis, lowering the post-prandial glycaemic peak, and exerting their anti-diabetic effect [13]. Furthermore, phenolic compounds may inhibit the proliferation of gastric and colon cancer cells by delaying the progression of gastric and colon–rectal cancers [8,14]. Moreover, phenolic compounds may behave as antioxidants in the gastro-intestinal tract, limiting and delaying lipid oxidation in meat and protecting the body from the toxic action of lipid hydroperoxides and advanced lipoxidation end-products [15]. Finally, dietary phenolic compounds may change the gut microbiota composition by reducing the community of bacteria involved in the onset of colon cancer and inflammation and increasing that of health-promoting bacteria [9,10]. Therefore, the gastro-intestinal tract can be seen as one of the major sites for the biological action of phenolic compounds.

To fulfil their effect in the gastro-intestinal tract, phenolic compounds need to be released from the food matrix during digestion and have to be stable in the gastro-intestinal environment. Gastro-intestinal release and stability of phenolic compounds are included in the definition of bioaccessibility, described as the fraction of phenolic compounds available for absorption in the intestine [16,17]. Phenolic compounds bioaccessibility is primarily influenced by the food matrix, the chemical structure of phenolic compounds, the chemical conditions in the gastro-intestinal tract (i.e., presence of proteins, bile salts, and the pH), as well as the enzymatic activity of digestive enzymes [18,19,20].

Since in vivo studies involving human subjects are time-consuming, tedious, and expensive, in vitro models have been preferentially used for estimating phenolic compounds bioaccessibility in several fruits and beverages [16,21]. The strength of in vitro gastro-intestinal digestion models is related to their simplicity, reproducibility, and robustness [16,21]. Next, the development of the INFOGEST protocol made it possible to harmonize the experimental conditions and to develop a model that applies conditions as closely as possible to physiological ones [22]. Although several studies have been carried out in recent years on the bioaccessibility of phenolic compounds from vegetable foods [21], there actually is a lack of published articles aimed at comparing the bioaccessibility of specific classes of phenolic compounds in the function of their chemical structure and the food matrix. Therefore, this study was designed to analyse the in vitro bioaccessibility of three classes of phenolic compounds (flavones, flavanones, and flavonols) from selected vegetable foods and beverages to shed light on the influence of the chemical structure and food matrix on the in vitro bioaccessibility of phenolic compounds.

## 2. Materials and Methods

### 2.1. Materials

The digestive enzymes (human salivary α-amylase, porcine pepsin, and porcine pancreatin) and chemicals for the in vitro gastro-intestinal digestion procedure were purchased from Sigma (Milan, Italy). Solvents for phenolic compounds extraction and high-resolution mass spectrometry analysis were obtained from BioRad (Hercules, CA, USA). Vegetable foods and beverages (blonde orange, blood orange “Tarocco”, blonde orange juice 100%, rooibos leaves, chamomile flowers, green tea leaves, red radicchio, capers, and red-skinned onion) were purchased in a local supermarket (Reggio Emilia, Italy). The standards of phenolic compounds used for mass spectrometry quantification are listed in Supplementary Table S1.

### 2.2. Preparation of Vegetable Foods and Beverages

Capers and red radicchio were appropriately washed with distilled water, dried on absorbent paper, and stored at −80 °C until use. Red-skinned onion, blonde orange, and blood orange were peeled, washed, and dried on absorbent paper before storage at −80 °C. Chamomile tea and rooibos tea were prepared according to the typical homemade preparation. Briefly, 1.5 g of chamomile flowers, or 2 g of rooibos tea leaves, or 1.3 g of green tea leaves were left in infusion with 100 mL of boiling water for 5 min before removal. Beverages were then stored at −80 °C until analysis. Blonde orange juice was stored at −80 °C as such.

### 2.3. Extraction of Phenolic Compounds from Vegetable Foods and Beverages

Phenolic compounds from vegetables (blonde orange, blood orange, red radicchio, capers, and red-skinned onion) were extracted as described in Nissen et al. [10]. Briefly, 15 g of food were mixed with 30 mL of a water/methanol/formic acid (28:70:2, *v*/*v*/*v*) solution, homogenized with an Ultra-Turrax, and then incubated at 37° C for 30 min under stirring. Then, the extracts were centrifuged at 6000× *g* (20 min; 4 °C), and the supernatants were withdrawn and stored at −80 °C before the injection in the high-resolution mass spectrometer for phenolic compounds identification and quantification. Beverages (blonde orange juice, rooibos tea, green tea, and chamomile tea) were directly injected into the high-resolution mass spectrometer after centrifugation (10,000× *g*; 20 min; 4 °C).

### 2.4. In Vitro Gastro-Intestinal Digestion of Vegetable Foods and Beverages

In vitro gastro-intestinal digestion was performed exactly as reported in the INFOGEST 2.0 protocol [22]. The entire digestive process took 242 min. Briefly, 10 g of vegetable foods were mixed with 10 mL of salivary fluids, homogenized with a pestle and mortar to mimic mastication, and then incubated at 37 °C for 2 min in a rotating wheel (10 rpm) after the addition of salivary α-amylase (150 U/mL of final concentration in the digestive system). In the case of beverages, 10 mL of salivary fluids were added to 10 mL of beverage and incubated as reported above after the addition of salivary α-amylase. Next, to simulate the gastric phase of the digestion, 20 mL of gastric fluid were mixed with the bolus, and pepsin was added at 2000 U/mL (final concentration in the digestive system) after bringing the pH to 3 with 6 mol/L HCl. The bolus was then incubated at 37 °C in a rotating wheel (10 rpm) for 120 min. At the end of the gastric phase of the digestion, the bolus was mixed with 40 mL of intestinal fluid, and the pH was brought to 7.5 with concentrated NaOH. The chyme was incubated for 30 min at 37 °C in a rotating wheel (10 rpm) before the addition of pancreatin (final concentration in the digestive system based on trypsin activity of 200 U/mL). The intestinal phase of the digestion was then simulated by incubating the chyme in a rotating wheel (10 rpm) at 37 °C for 120 min.

At the end of the digestion, the samples were centrifuged (10,000× *g*; 20 min; 4 °C) and stored at −80 °C before the injection into the high-resolution mass spectrometer.

### 2.5. Identification and Quantification of Phenolic Compounds by High-Resolution Mass Spectrometry

High-resolution mass spectrometry analysis was carried out by using a Q Exactive Hybrid Quadrupole-Orbitrap Mass Spectrometer coupled to a UHPLC Ultimate 3000 module (Thermo Fisher Scientific, San Jose, CA, USA). Phenolic compounds were separated by using a C18 column (Acquity UPLC HSS C18 Reversed phase, 2.1 mm × 100 mm, 1.8 µm particle size, Waters, Milan, Italy) with a binary gradient of water (containing the 0.1% of formic acid) and acetonitrile (containing the 0.1% of formic acid. The gradient, the flow rate, and all the other chromatographic and mass spectrometry conditions are described in Martini et al. [23]. Phenolic compound quantification was performed by building external calibration curves with the available standards as reported in Supplementary Table S1. Results are reported as mg/100 g of food or beverage. In the case of chamomile, rooibos, and green teas, the results are reported as mg/100 mL of beverage. The bioaccessibility index (BI) was calculated as described in Cattivelli et al. [24]. The mass spectrometry data of the identified phenolic compounds are reported in Supplementary Table S1.

### 2.6. Statistics

In vitro digestion and phenolic compound extraction were performed in triplicate for each sample. Data are presented as mean ± SD for three injections for each prepared sample. Differences among samples were calculated by one-way ANOVA (univariate analysis of variance) with Tukey’s post hoc test by using Graph Pad Prism 9.0 (GraphPad Software, San Diego, CA, USA). The differences were considered significant with *p* <0.05.

## 3. Results

### 3.1. Identification and Quantification of Flavanones, Flavones, and Flavonols in Vegetable Foods and Beverages

#### 3.1.1. Identification and Quantification of Flavanones in Blonde Orange, Blood Orange, Blonde Orange Juice, and Rooibos Tea

The flavanone profile of blonde orange, blood orange, and blonde orange juice was quite similar, with only some quantitative differences (see Supplementary Table S2 for the complete flavanone profiles of orange samples).

**Figure 1:**
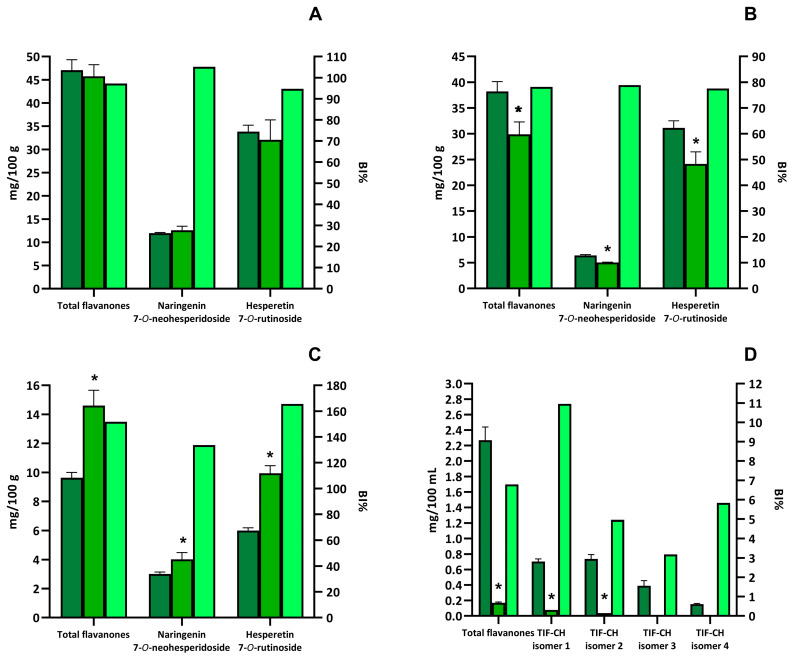
Quantification of total and individual flavanones in selected beverages and vegetable foods and bioaccessibility index (BI%). Dark green bars represent the amount of total or individual flavanones detected in the extracts. Green bars represent the amount of total or individual flavanones detected in the digested sample. Light green bars represent the bioaccessibility index for total or individual flavanones. The amount of total or individual flavanones is expressed as mg/100 g or mg/100 mL and reported in the left y-axis. The bioaccessibility index is expressed as the percentage of remaining compound after digestion, with respect to the amount in the extract, and is reported in the left x-axis. Selected foods were blonde orange (**A**), blood orange (**B**), orange juice (**C**), and rooibos tea (**D**). Asterisks indicate that the value is significantly (*p* < 0.05) different, with respect to the undigested sample. TIF-CH means tetra-hydroxyflavanone-C-hexoside.

The highest total flavanones content was found in the blonde orange (47.08 ± 2.25 mg/100 g of fruit), followed by the blood orange (38.22 ± 1.91 mg/100 g of fruit) and blonde orange juice (9.63 ± 0.37 mg/100 g of juice) (Figure 1A–C). In all the orange samples, the flavanone profiles were dominated by O-glycosylated derivatives of naringenin and hesperetin. The compounds found in the highest amount were hesperetin-7-O-rutinoside and naringenin-7-O-neohesperidoside. These two compounds accounted for 97.3%, 98.2%, and 93.6% of total flavanones in blonde orange, blood orange, and blonde orange juice, respectively (Figure 1A–C).

Rooibos tea contained a lower content of flavanones (2.47 ± 0.15 mg/100 mL of beverage) than the orange samples (Figure 1D). Moreover, the rooibos tea flavanones profile was diverse from orange samples, mainly containing C-glycosylated derivatives (Supplementary Table S2). The compounds present in the highest amount were four isomers of tetra-hydroxy-flavanone-C-hexoside, representing 89.0% of total flavanones (Figure 1D).

#### 3.1.2. Identification and Quantification of Flavones in Red Radicchio, Chamomile Tea, Rooibos Tea, and Green Tea

**Figure 2:**
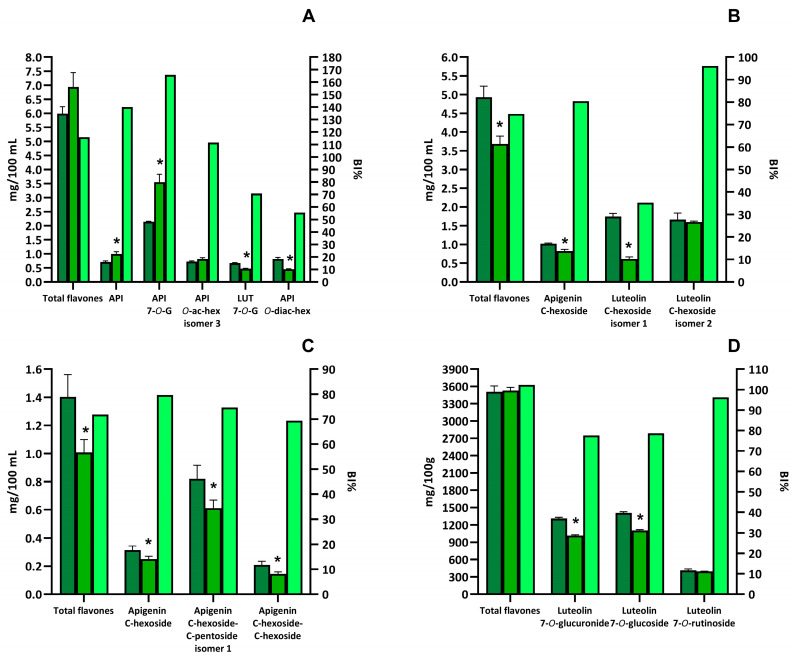
Quantification of total and individual flavones in selected beverages and vegetable foods and bioaccessibility index (BI%). Dark green bars represent the amount of total or individual flavones detected in the extracts. Green bars represent the amount of total or individual flavones detected in the digested sample. Light green bars represent the bioaccessibility index for total or individual flavones. The amount of total or individual flavones is expressed as mg/100 g or mg/100 mL and is reported in the left y-axis. The bioaccessibility index is expressed as the percentage of remaining compound after digestion respect to the amount in the extract and is reported in the left x-axis. Selected foods were chamomile tea (**A**), rooibos tea (**B**), green tea (**C**), and red radicchio (**D**). Asterisks indicate that the value is significantly (*p* < 0.05) different, with respect to the undigested sample. API means apigenin whereas LUT means luteolin.

The entire flavone profiles of the selected samples are shown in Supplementary Table S3. The highest amount of flavones was found in red radicchio (3447.72 ± 102.13 mg/100 g of red radicchio), followed by chamomile tea (5.99 ± 0.21 mg/100 mL of beverage), rooibos tea (4.93 ± 0.30 mg/100 mL of beverage), and green tea (1.44 ± 0.16 mg/100 mL of beverage) (Figure 2). Red radicchio mainly contained O-glycosylated luteolin derivatives, with a minor amount of glycosylated apigenin derivatives. The most important compounds from a quantitative point-of view were luteolin-7-O-glucoside, luteolin-7-O-glucuronide, and luteolin-7-O-rutinoside. These three flavones accounted for 90.8% of the total flavones in red radicchio (Figure 2D).

Differently from red radicchio, chamomile tea contained mainly O-glycosylated derivatives of apigenin (Supplementary Table S3 and Figure 2A). The main compound identified in chamomile tea was apigenin-7-O-glucoside, which accounted for 35.7% of total flavones. Appreciable amounts of different isomers of apigenin-O-acetyl-hexoside, one isomer of apigenin-O-diacetyl-hexoside, and apigenin aglycone were also present. The most important luteolin derivative was luteolin-7-O-glucoside (Figure 2A).

Rooibos tea was also a major source of flavones (Supplementary Table S3). In this case, the phenolic profile was dominated by C-glycosylated derivatives of both luteolin and apigenin. In particular, two isomers of luteolin-C-hexoside accounted for 69.2% of total flavones, whereas one isomer of apigenin-C-hexoside for 20.7% (Figure 2B).

Like rooibos tea, green tea also exclusively contained C-glycosylated flavones (Supplementary Table S3). The compound present at the highest concentration was an isomer of apigenin-C-hexoside-C-pentoside, which represented 58.5% of the total flavones in green tea. Lower amounts of apigenin-C-hexoside and apigenin-C-hexoside-C-hexoside were also detected (Figure 2C).

#### 3.1.3. Identification and Quantification of Flavonols in Capers, Red-Skinned Onion, Chamomile Tea, Rooibos Tea and Green Tea

**Figure 3:**
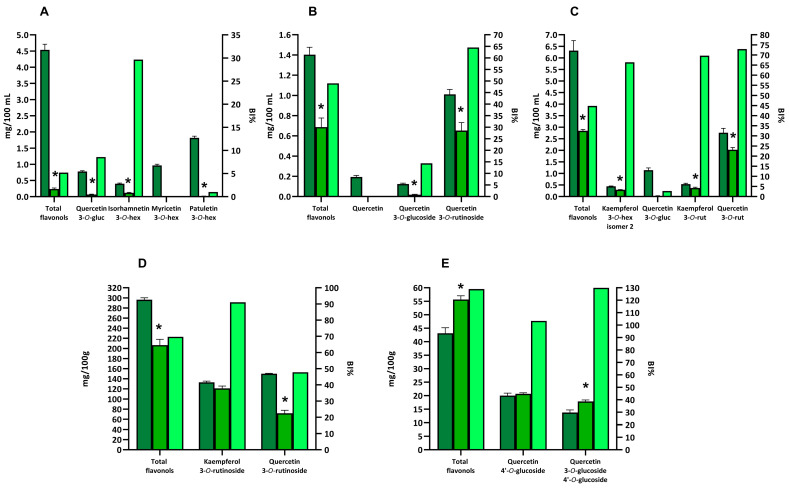
Quantification of total and individual flavonols in selected beverages and vegetable foods and bioaccessibility index (BI%). Dark green bars represent the amount of total or individual flavonols detected in the extracts. Green bars represent the amount of total or individual flavonols detected in the digested sample. Light green bars represent the bioaccessibility index for total or individual flavonols. The amount of total or individual flavonols is expressed as mg/100 g or mg/100 mL and is reported in the left y-axis. The bioaccessibility index is expressed as the percentage of the remaining compound after digestion, with respect to the amount in the extract, and is reported in the left x-axis. Selected foods were chamomile tea (**A**), rooibos tea (**B**), green tea (**C**), capers (**D**), and red-skinned onion (**E**). Asterisks indicate that the value is significantly (*p* < 0.05) different, with respect to the undigested sample.

The complete flavonol profiles of the selected samples are shown in Supplementary Table S4. Among the tested products, caper was the one with the highest quantity of total flavonols (296.28 ± 3.56 mg/100 g of capers), followed by red-skinned onion (43.13 ± 2.00 mg/100 g of onion), green tea (6.48 ± 0.44 mg/100 mL of beverage), chamomile tea (4.54 ± 0.17 mg/100 mL of beverage), and rooibos tea (1.40 ± 0.07 mg/100 mL of beverage) (Figure 3).

Green tea contained both quercetin and kaempferol 3-O-glycosylated derivatives, mainly quercetin-3-O-rutinoside, quercetin-3-O-glucoside, kampferol-3-O-rutinoside, and kaempferol-3-O-hexoside (Supplementary Table S4). In particular, quercetin derivatives were present in higher concentrations compared to kaempferol derivatives with quercetin-3-O-rutinoside and quercetin-3-O-glucoside that accounted for 43.7% and 18.1% of total flavonols (Figure 3C). Instead, the two main kaempferol derivatives, kampferol-3-O-rutinoside and kaempferol-3-O-hexoside, represented the 8.5% and 7.0% of total flavonols. Furthermore, some additional quercetin and kaempferol mono-, di-, and tri-glycosides were also identified, although at a lower concentration (Supplementary Table S4).

Chamomile tea was characterized by the presence mainly of 3-O-mono-glycosylated derivatives of flavonols (Supplementary Table S4). The compounds present at the highest concentrations were the O-methylated flavonol patuletin-3-O-glucoside (accounting for 40.1% of total flavonols), myricetin-3-O-glucoside (accounting for 21.3% of total flavonols), quercetin-3-O-glucoside (accounting for 17.2% of total flavonols), and isorhamnetin-3-O-glucoside (accounting for 8.8% of total flavonols) (Figure 3A).

The flavonol profile of capers was dominated by the 3-O-rutinoside derivatives of quercetin and kaempferol (Supplementary Table S4). These two compounds were present in similar amounts, accounting for 95.5% of total flavonols (Figure 3D).

Quercetin-3-O-rutinoside was also the main flavonol identified in rooibos tea, representing 72.1% of total flavonols (Figure 3B). A minor amount of additional quercetin-3-O-glycosylated derivatives and quercetin aglycone was also detected (Figure 3B).

Finally, the flavonol profile of the red-skinned onion was quite different since the major flavonols were 4′-O derivatives of quercetin (Supplementary Table S4). The most important flavonols from a quantitative point-of-view were quercetin-4′-O-glucoside and quecetin-3-O-glucoside-4′-O-glucoside, since they represented 46.4% and 31.9% of total flavonols, respectively (Figure 3E). In addition, an appreciable amount of isorhamnetin-4′-O-glucoside was also detected (Supplementary Table S4).

### 3.2. Bioaccessibility of Flavanones from Selected Vegetable Foods and Beverages

Orange O-glycosylated flavanones were easily extracted from the food matrices and appeared to be quite stable during in vitro gastro-intestinal digestion of orange fruits and juice (Supplementary Table S5).

In the blonde orange fruit, the bioaccessibility index of total flavanones as well as of hesperetin-7-O-rutinoside and naringenin-7-O-neoesperidoside was near to or higher than 100% (Figure 1A).

A slightly lower bioaccessibility index was found for total flavanones, hesperetin-7-O-rutinoside, and naringenin-7-O-neoesperidoside after in vitro gastro-intestinal digestion of blood orange (Figure 1B). A bioaccessibility index of 151.7% was found for total flavanones in blonde orange juice (Figure 1C). The increase in total flavanone concentrations was entirely due to an augment in hesperetin-7-O-rutinoside and naringenin-7-O-neoesperidoside concentrations (Figure 1C).

Diversely from the O-glycosylated orange flavanones, the C-glycosylated forms found in rooibos tea were characterized by a very low bioaccessibility (Supplementary Table S4). As reported in Figure 1D, the rooibos tea total flavanones bioaccessibility index was 11.3%. This low bioaccessibility index was mainly due to the degradation of the main flavanones found in rooibos tea, i.e., four isomers of tetra-hydroxy-flavanone-C-hexoside, during in vitro gastro-intestinal digestion (Figure 1D).

### 3.3. Bioaccessibility of Flavones from Selected Vegetable Foods and Beverages

Generally, total flavone bioaccessibility was very high both in beverages and red radicchio (Supplementary Table S6).

The highest bioaccessibility values were found for chamomile tea (116.0%) and red radicchio (102.4%) (Figure 2A,D). On the contrary, rooibos and green teas mainly contained C-glycosylated flavones, which appeared to be less stable than the corresponding O-glycosylated forms, with a bioaccessibility index for total flavones of 74.7% and 71.8% in rooibos tea and green tea, respectively (Figure 2B,C and Supplementary Table S6).

Apigenin-O-glycosylated derivatives mainly characterized the flavone profile of chamomile tea. All these compounds were characterized by a high bioaccessibility index, sometimes higher than 100% (Figure 2A).

By looking at the individual compound, apigenin-7-O-glucoside (the main compound found in chamomile tea) was characterized by the highest bioaccessibility of 165.9% (Figure 2A). Similarly, during in vitro digestion of chamomile tea, a bioaccessibility index above 100% was found for apigenin (Supplementary Table S6).

Differently from chamomile tea, rooibos and green teas mainly contained C-glycosylated derivatives of both apigenin and luteolin. These derivatives were characterized by a lower, although still high, bioaccessibility compared to the O-glycosylated derivatives (Supplementary Table S6 and Figure 2B,C). For example, apigenin-C-hexoside bioaccessibility was 80.5% in rooibos tea and 79.7% in green tea (Figure 2B,C). Similarly, the bioaccessibility of four isomers of apigenin-C-hexoside-C-pentoside ranged between 74.7 and 93.4%, whereas that of apigenin-C-hexoside-C-hexoside ranged from 68.4% to 90.2% (Supplementary Table S6 and Figure 2B,C).

Red radicchio, the only solid food containing flavones tested in the study, was particularly rich in luteolin-O-glycosylated derivatives (Supplementary Table S6 and Figure 2D). During in vitro digestion of red radicchio, flavones were easily released from the food matrix and appeared to be stable under gastro-intestinal conditions.

### 3.4. Bioaccessibility of Flavonols from Selected Vegetable Foods and Beverages

The bioaccessibility of total flavonols in the digested beverages was quite low, being 5.2%, 44.8%, and 49.0% in chamomile tea, green tea, and rooibos tea, respectively, compared to flavanones and flavones (Supplementary Table S7 and Figure 3A–C).

Chamomile tea, the beverage with the lowest bioaccessibility index, mainly contained patuletin-3-O-glucoside, myricetin-3-O-glucoside, and quercetin-3-O-glucoside, all compounds characterized by a very low bioaccessibility index (Figure 3A). Very low bioaccessibility values were observed for quercetin-3-O-glucoside also after in vitro digestion of green tea and rooibos tea (Figure 3B,C).

Rooibos and green teas displayed a higher bioaccessibility index for flavonols than chamomile tea (Figure 3B,C). These two beverages mainly contained quercetin-3-O-rutinoside, which was found to be more stable than quercetin-3-O-glucoside during in vitro digestion. The bioaccessibility index of quercetin-3-O-rutinoside was 64.5% and 72.9% in rooibos and green teas, respectively.

Green tea was also rich in kaempferol derivatives, especially kaempferol-3-O-hexoside and kaempferol-3-O-rutinoside (Figure 3C). Compared to the corresponding glycosylated quercetin, kaempferol-3-O-hexoside was found to be more stable under gastro-intestinal conditions with a bioaccessibility index of 66.4%. Similarly, kaempferol-3-O-rutinoside was characterized in green tea for a high bioaccessibility index (69.7%).

The bioaccessibility index of total flavonols for capers was 69.7%, significantly higher than that of the different beverages (Figure 3D). Capers were rich in rutinoside derivatives of quercetin and kaempferol that were easily released from the food matrix during digestion and stable during in vitro gastro-intestinal digestion with a bioaccessibility index of 91.0% and 47.8% for kaempferol-3-O-ruitnoside and quercetin-3-O-rutinoside, respectively.

The total flavonols bioaccessibility index was above 100% in red-skinned onion (Figure 3E). The main compounds identified in red-skinned onion were quercetin-4′-O-glucoside and quercetin-3-O-glucoside-4′-O-glucoside, displaying a bioaccessibility index of 103.3% and 130.0%, respectively.

## 4. Discussion

Phenolic compounds have to be bioavailable to exert their effect at the systemic level and/or in the gastro-intestinal tract [2,3]. The bioavailability of phenolic compounds depends on several factors, including the food matrix, the release from the food matrix, their stability under gastro-intestinal conditions, and the passage across the intestinal mucosa [2,3]. The study of the bioaccessibility of phenolic compounds is fundamental because it allows for understanding the efficiency with which these compounds are released from the food matrix and their stability in the gastrointestinal environment. In this study, selected beverages and foods rich in three different classes of flavonoids (flavanones, flavones, and flavonols) have been digested in vitro to investigate their bioaccessibility using the INFOGEST protocol.

Two solid foods, blonde orange and blood orange, and two beverages, blonde orange juice and rooibos tea, were selected as flavanone-rich foods. The flavanone profiles of the three orange-based products were comparable from a qualitative point-of-view, with hesperetin-7-O-rutinoside and naringenin-7-O-neohesperidoside being the major flavanones identified. In general, the O-glycosylated derivatives of flavanones dominated the flavanone profiles of these products. These results agree with previously reported data on flavanones in orange and orange juice [25,26,27]. From a quantitative point-of-view blonde orange and blood orange contained a higher content of flavanones than orange juice. According to previous studies, orange juice contained fewer flavanones than orange fruit [28]. Indeed, a part of the extracted flavanones is subjected to precipitation by self-aggregation or after binding with macromolecules during the processing of orange juice [29]. Moreover, flavanones in orange fruit are predominantly located in the albedo and membranes, which are separated from the juice during the processing stage [28]. The overall bioaccessibility of total flavanones and individual O-glycosylated flavanones in blonde orange was near 100%, whereas it was below 80% in blood orange. The only difference between blonde orange and blood orange is related to the presence of anthocyanins in the latter fruit [30]. The presence of anthocyanins in blood orange may have impacted the bioaccessibility of flavanones. It is well-known that anthocyanins may react under acidic and slightly alkaline conditions (such as in the gastric and intestinal fluids, respectively) with other flavonoids (such as flavanols), giving dimeric adducts [31,32]. In orange juice, the bioaccessibility index of total flavanones as well as of hesperetin-7-O-rutinoside and naringenin-7-O-neohesperidoside was above 100%, suggesting a release of these compounds from the juice during in vitro gastro-intestinal digestion. The composition of the gastrointestinal environment and the presence of digestive enzymes likely played a role in either resuspending the flavanones that had precipitated during juice preparation or in hydrolysing macromolecules, which would lead to the release of flavanones that were previously bound to them. Moreover, the flavanones can also be released from albedo during the digestion. Similar stability of orange O-glycosylated flavanones during in vitro gastro-intestinal digestion has already been reported both in fruit and with pure compounds [18,20,26,33]. Contrarily, Aschoff et al. [28] found a lower bioaccessibility index for orange flavanones during the digestion of the fruit. They attributed this low bioaccessibility to a possible saturation effect of flavanones in the digestive fluid. However, their orange sample presented a flavanones concentration more than 5 times higher than the orange fruit used in this study.

Differently from the orange-based products, rooibos tea mainly contained C-glycosylated flavanones. The bioaccessibility of C-glycosylated flavanones was very low (<11%) compared to the O-glycosylated ones, suggesting that C-glycosylation of flavanones had a thorough impact on their bioaccessibility. No previous studies regarding the bioaccessibility of C-glycosylated flavanones are present in the literature.

Three beverages (chamomile tea, rooibos tea, and green tea) as well as a solid food (red radicchio) were selected as flavone-rich foods. Red radicchio showed the highest amount of flavones, primarily comprised of O-glycosylated luteolin derivatives. Very few studies have been carried out o identify and quantify the flavone profile of red radicchio. Carazzone et al. [34] identified only one flavone (apigenin-7-O-glucoside) in red radicchio, whereas Cefola et al. [35] found luteolin-7-O-glucuronide as the main flavone in red radicchio. Similarly, the flavones profile of chamomile tea was dominated by O-glycosylated flavones, especially apigenin derivatives. The reported data agree with previous studies that identified apigenin derivatives as the major flavones in chamomile flowers or tea [36,37]. Differently, rooibos tea and green tea contained mainly C-glycosylated derivatives of flavones. Previously, C-glycosylated derivatives of both luteolin and apigenin have been identified as the main flavones in rooibos leaves or tea [38,39,40]. Moreover, apigenin-C-hexoside-C-pentoside and apigenin-C-hexoside have been already identified in green tea [41,42].

Flavones in chamomile tea and red radicchio were characterised by a very high bioaccessibility (about 100%). These two foods contained mainly O-glycosylated flavones, which, similarly to what was observed for flavanones, were stable under gastro-intestinal conditions. This indicated that the O-glycosylated derivatives of apigenin and luteolin were very stable under gastro-intestinal conditions. Apigenin-7-O-glucoside was the primary compound found in chamomile tea, exhibiting a bioaccessibility index exceeding 100%. The increase in apigenin-7-O-glucoside concentration after in vitro gastro-intestinal digestion may be a consequence of the hydrolysis of apigenin-O-di-hexoside, as suggested by the bioaccessibility index below 100% of this compound. Similar behaviour has been already observed during the in vitro digestion of onion where quercetin-di-hexosides were hydrolysed to quercetin-mono-hexosides [43]. Hanske et al. [44] in vitro digested pure apigenin-7-O-glucoside finding a bioaccessibility index of about 80% at the end of the intestinal phase of digestion. The authors also revealed that part of apigenin-7-O-glucoside was de-glycosylated to apigenin in the intestinal milieu. Similarly, during in vitro digestion of chamomile tea, apigenin demonstrated a bioaccessibility index greater than 100%. Probably, the increase in apigenin concentration after in vitro digestion of chamomile tea may be due to the hydrolysis of apigenin-7-O-glucoside to apigenin. Several previous studies indicated that pure apigenin remained highly stable during in vitro digestion [18,45]. Differently from chamomile tea and red radicchio, rooibos and green teas mainly contained C-glycosylated derivatives of both apigenin and luteolin. These derivatives were characterized by a lower, although still high, bioaccessibility compared to the O-glycosylated derivatives. Flavone-C-glycosides have been already found resistant to gastro-intestinal conditions [41,46,47]. Regarding the aglycones, apigenin (overall bioaccessibility considering both chamomile and rooibos teas of 137.7%) was more stable than luteolin (overall bioaccessibility considering both chamomile and rooibos teas of 69.3%). The catechol moiety in the B-ring of luteolin may have impacted its stability because this structural motif is more prone to oxidation in alkaline intestinal conditions [48]. Methylation of one hydroxyl group of luteolin, as observed in tri-hydroxy-methoxyflavones, increased the molecule stability (overall bioaccessibility considering both chamomile and rooibos teas of 99.5%). During in vitro digestion of red radicchio, flavones were readily released from the food matrix and appeared stable under gastro-intestinal conditions. An increase in the concentration of the aglycone luteolin was observed, which may be related to the hydrolysis of luteolin-7-O-glucoside, whose concentration decreased by about 21% during digestion.

Three beverages (chamomile tea, rooibos tea, and green tea) and two solid foods (caper and red-skinned onion) were selected as flavonols-rich foods. The flavonols profiles of the different studied foods agreed with previously reported data [38,39,40,43,49,50,51,52,53,54]. Among flavonols, the 3-O-glucoside derivatives were characterized by a low bioaccessibility index. Previous studies reported the instability of quercetin-3-O-glucoside and myricetin-3-O-glucoside during the in vitro digestion of beverages or fruit extracts [19,41,54]. These compounds were characterized by a catechol moiety in their B-ring, which negatively impacted flavonol stability under alkaline intestinal conditions, as described above [48]. Alternatively, the mentioned 3-O-glucoside derivatives may undergo hydrolysis of the glycosidic bond. Previous studies demonstrated that quercetin-3-O-glucoside was hydrolysed to the quercetin aglycone during in vitro digestion of onion, apple, or pure compounds [47,55,56]. Recently, Cattivelli et al. [24] found that both oxidative degradation and hydrolysis reactions of glycosylated quercetin derivatives occurred during in vitro digestion of onion. Differently, quercetin-3-O-rutinoside showed more stability than the corresponding 3-O-glucoside. Previously, quercetin-3-O-rutinoside was found to be stable during in vitro digestion of green tea or pure compound [41,57]. Therefore, the presence of a rutinoside moiety seemed to confer more stability during in vitro digestion to the quercetin derivatives compared to the presence of a glucose group. Furthermore, kaempferol-3-O-glucoside, found in green tea, appeared more stable than the corresponding quercetin-3-O-glucoside. Previously, kaempferol glycosides have been found to be more stable than the corresponding quercetin and myricetin glycosides [41]. The higher stability of kaempferol derivatives under gastro-intestinal conditions may be due to their low oxidation rate under alkaline conditions as a consequence of the lack of the catechol moiety in the B-ring [58]. Regarding flavonols, a significant food matrix effect was noted when comparing the bioaccessibility indexes of digested solid foods (caper and red-skinned onion) with those of beverages. Moreover, the mono-hexoside quercetin-4′-O-glucoside found in red-skinned onion was considerably more stable than the corresponding mono-hexoside quercetin-3-O-glucoside detected in beverages. This higher stability may be due to the food matrix effect and to the glucoside moiety in position 4′ in the B-ring, which masks the catechol group, making this compound less susceptible to oxidative degradation.

**Figure 4:**
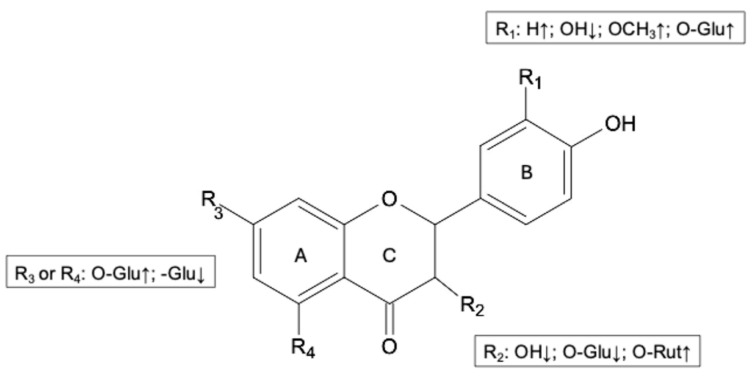
Structural characteristics of functional groups that affect the bioaccessibility of flavanones, flavones, and flavonols. ↑ indicates high bioaccessibility index; ↓ indicates low bioaccessibility index; Glu means a glucoside moiety; Rut means a rutinoside moiety.

Figure 4 shows a summary of the structural characteristics of flavanones, flavones, and flavonols that impact their bioaccessibility.

The presence of an -OH group in R_1_, as observed in luteolin derivatives and quercetin derivatives, negatively impacted the bioaccessibility index compared to apigenin derivatives and kaempferol derivatives that lack the -OH group. An increase in bioaccessibility index was also ascertained when the -OH group was linked with a methyl group or a glucose moiety, as noted in tri-hydroxy-methoxy flavones and quercetin-4′-O-glucoside, respectively. Moreover, the presence of an -OH group and an -O-glucoside group in the R_2_ position caused a decrease in the bioaccessibility index as assessed by comparing the gastro-intestinal stability of flavonols with their corresponding flavones and flavanones. On the contrary, an -O-rutinoside group positively influenced flavonol bioaccessibility. Regarding the A-ring, the presence of an -O-glucoside group instead of a -C-glucoside group enhanced the gastro-intestinal stability of flavones and flavanones.

## 5. Conclusions

The different classes of studied flavonoids presented different bioaccessibility indexes depending on their structure. Flavanones and flavones, especially the O-glycosylated derivatives, were characterized by great stability under gastro-intestinal conditions. Furthermore, C-glycosylation of flavones and flavanones decreased the bioaccessibility index compared to the O-glycosylated compound. Flavonols were found to be less stable, and increased flavonol B-ring hydroxylation was related to reduced stability, as observed in the case of kaempferol and quercetin derivatives. These results suggest that a catechol moiety in the B-ring negatively affected the gastro-intestinal stability of flavonoids. Moreover, flavonoids were easily released from solid food matrices, suggesting the gastro-intestinal tract may act as a bio-extractor, and the food matrix may sometimes protect flavonoids from degradation. This study may help to reach a better understanding of the potential beneficial effects of flavonoids especially at the gastro-intestinal level.

## Data Availability

The original contributions presented in the study are included in the article/Supplementary Material; further inquiries can be directed to the corresponding author.

## Supplementary Materials

The following supporting information can be downloaded at https://www.mdpi.com/article/10.3390/biology13121081/s1: Table S1: Mass spectrometry data of flavanones, flavones and flavonols identified in the different beverages and vegetable foods and characteristic of the standard compounds used for quantification. Table S2: Amount of flavanones in selected beverages and vegetable foods before digestion. Table S3: Amount of flavones in selected beverages and vegetable foods before digestion. Table S4: Amount of flavonols in selected beverages and vegetable foods. Table S5: Amount of flavanones in selected beverages and vegetable foods after in vitro gastro-intestinal digestion. Table S6: Amount of flavones in selected beverages and vegetable foods after in vitro gastro-intestinal digestion. Table S7: Amount of flavonols in selected beverages and vegetable foods after in vitro gastro-intestinal digestion.
