# Supplementary material for: Bioaccessibility of Flavones, Flavanones, and Flavonols from Vegetable Foods and Beverages
Source: Biology (Basel). 2024 Dec 22;13(12):1081. doi: 10.3390/biology13121081 (PMC11672976; doi:10.3390/biology13121081)
Supplement: Supplementary file 1 [file biology-13-01081-s001.zip › Supplementary Table S2.pdf]

**Supplementary Table S2.** Amount of flavanones in selected beverages and vegetable foods before digestion. Results are expressed in mg of flavanones/100g or 100 mL of vegetable foods or beverages.

| Compound | Blonde Orange | Blood Orange | Orange Juice | Rooibos |
|---|---|---|---|---|
| Tri-hydroxy-flavanone | n.d. | n.d. | 0.005 ± 0.000 | n.d. |
| Naringenin | n.d. | n.d. | 0.002 ± 0.000 | 0.007 ± 0.000 |
| Tetra-hydroxy-flavanone | n.d. | n.d. | n.d. | 0.037 ± 0.002 |
| Naringenin-C-hexoside isomer 1 | n.d. | n.d. | n.d. | 0.015 ± 0.000 |
| Naringenin-C-hexoside isomer 2 | n.d. | n.d. | n.d. | 0.015 ± 0.001 |
| Naringenin-C-hexoside isomer 3 | n.d. | n.d. | n.d. | 0.016 ± 0.001 |
| Naringenin-*O*-hexoside | 0.214 ± 0.030 | 0.028 ± 0.01 | 0.062 ± 0.007 | n.d. |
| Tetra-hydroxyflavanone-C-hexoside isomer 1 | n.d. | n.d. | n.d. | 0.703 ± 0.034 |
| Tetra-hydroxyflavanone-C-hexoside isomer 2 | n.d. | n.d. | n.d. | 0.736 ± 0.057 |
| Tetra-hydroxyflavanone-C-hexoside isomer 3 | n.d. | n.d. | n.d. | 0.389 ± 0.067 |
| Tetra-hydroxyflavanone-C-hexoside isomer 4 | n.d. | n.d. | n.d. | 0.153 ± 0.009 |
| Tetra-hydroxyflavanone-*O*-hexoside isomer 1 | 0.019 ± 0.000 | 0.015 ± 0.003 | 0.009 ± 0.000 | n.d. |
| Tetra-hydroxyflavanone-*O*-hexoside isomer 2 | 0.012 ± 0.001 | 0.014 ± 0.002 | n.d. | n.d. |
| Penta-hydroxyflavanone-*O*-hexoside isomer 1 | n.d. | 0.030 ± 0.006 | n.d. | n.d. |
| Penta-hydroxyflavanone-*O*-hexoside isomer 2 | n.d. | 0.049 ± 0.008 | n.d. | n.d. |
| Naringenin-7-*O*-neohesperidoside | 11.970 ± 0.610 | 6.402 ± 0.475 | 3.005 ± 0.130 | n.d. |
| Naringenin-di-C-hexoside | n.d. | n.d. | n.d. | 0.015 ± 0.001 |
| Naringenin-*O*-hexoside-*O*-hexoside | 0.297 ± 0.017 | 0.175 ± 0.032 | 0.220 ± 0.005 | n.d. |
| Hesperetin-7-*O*-rutinoside | 33.843 ± 1.533 | 31.154 ± 1.348 | 6.004 ± 0.194 | n.d. |
| Tetra-hydroxyflavanone-di-C-hexoside | n.d. | n.d. | n.d. | 0.142 ± 0.007 |
| Narigenin-*O*-acetylhexoside-*O*-pentoside-pentoside | 0.107 ± 0.008 | 0.038 ± 0.005 | 0.028 ± 0.003 | n.d. |

| | | | | |
|---|---|---|---|---|
| Naringenin-*O*-hexoside-*O*-rutinoside | 0.449 ± 0.031 | 0.184 ± 0.015 | 0.136 ± 0.014 | n.d. |
| Hesperetin-*O*-rutinoside-*O*-hexoside | 0.169 ± 0.017 | 0.127 ± 0.013 | 0.158 ± 0.018 | n.d. |
| ***Total flavanones*** | ***47.079 ± 2.247*** | ***38.216 ± 1.907*** | ***9.629 ± 0.372*** | ***2.227 ± 0.169*** |

n.d. means that the compound was not detected in the sample.
