# Supplementary material for: Bioaccessibility of Flavones, Flavanones, and Flavonols from Vegetable Foods and Beverages
Source: Biology (Basel). 2024 Dec 22;13(12):1081. doi: 10.3390/biology13121081 (PMC11672976; doi:10.3390/biology13121081)
Supplement: Supplementary file 1 [file biology-13-01081-s001.zip › Supplementary Table S3.pdf]

**Supplementary Table S3.** Amount of flavones in selected beverages and vegetable foods before digestion. Results are expressed in mg of flavones/100g or 100 mL of vegetable foods or beverages.

| Compound | Chamomile | Rooibos | Green Tea | Red Radicchio |
|---|---|---|---|---|
| Apigenin | 0.709 ± 0.036 | 0.016 ± 0.001 | n.d. | n.d. |
| Luteolin | 0.012 ± 0.001 | 0.121 ± 0.004 | n.d. | 0.078 ± 0.003 |
| Tri-hydroxy-methoxyflavone isomer 1 | 0.039 ± 0.003 | 0.004 ± 0.000 | n.d. | n.d. |
| Tri-hydroxy-methoxyflavone isomer 2 | 0.011 ± 0.001 | n.d. | n.d. | n.d. |
| Tri-hydroxy-methoxyflavone isomer 3 | 0.007 ± 0.000 | n.d. | n.d. | n.d. |
| Tri-hydroxy-methoxyflavone isomer 4 | 0.010 ± 0.001 | 0.052 ± 0.004 | n.d. | n.d. |
| Apigenin-C-hexoside | n.d. | 1.020 ± 0.016 | 0.314 ± 0.029 | 15.709 ± 0.548 |
| Apigenin-7-*O*-glucoside | 2.140 ± 0.021 | n.d. | n.d. | 7.677 ± 0.180 |
| Apigenin-*O*-glucuronide | n.d. | n.d. | n.d. | 70.989 ± 5.110 |
| Luteolin-7-*O*-glucoside | 0.668 ± 0.015 | n.d. | n.d. | 1406.967 ± 19.369 |
| Luteolin-*O*-hexoside isomer 1 | n.d. | n.d. | n.d. | 24.882 ± 0.300 |
| Luteolin-*O*-hexoside isomer 2 | n.d. | n.d. | n.d. | 38.192 ± 1.679 |
| Luteolin-*O*-hexoside isomer 3 | n.d. | n.d. | n.d. | n.d. |
| Luteolin-C-hexoside isomer 1 | n.d. | 1.746 ± 0.080 | 0.032 ± 0.005 | n.d. |
| Luteolin-C-hexoside isomer 2 | n.d. | 1.664 ± 0.170 | 0.029 ± 0.024 | n.d. |
| Luteolin-C-hexoside isomer 3 | n.d. | n.d. | n.d. | 3.145 ± 0.036 |
| Luteolin-7-*O*-glucuronide | n.d. | n.d. | n.d. | 1312.577 ± 40.832 |
| Luteolin-*O*-glucuronide isomer 1 | n.d. | n.d. | n.d. | n.d. |
| Luteolin-*O*-glucuronide isomer 2 | n.d. | n.d. | n.d. | n.d. |
| Luteolin-*O*-glucuronide isomer 3 | n.d. | n.d. | n.d. | n.d. |
| Apigenin-*O*-acetylhexoside isomer 1 | 0.231 ± 0.013 | n.d. | n.d. | n.d. |
| Apigenin-*O*-acetylhexoside isomer 2 | 0.298 ± 0.022 | n.d. | n.d. | n.d. |
| Apigenin-*O*-acetylhexoside isomer 3 | 0.724 ± 0.024 | n.d. | n.d. | n.d. |
| Apigenin-*O*-diacetylhexoside | 0.817 ± 0.058 | n.d. | n.d. | n.d. |

| | | | | |
|---|---|---|---|---|
| Apigenin-*O*-malonylhexoside | 0.027 ± 0.001 | n.d. | n.d. | n.d. |
| Luteolin-*O*-malonylhexoside | 0.038 ± 0.002 | 0.013 ± 0.006 | n.d. | n.d. |
| Apigenin-C-hexoside-C-pentoside isomer 1 | 0.017 ± 0.001 | n.d. | 0.820 ± 0.096 | n.d. |
| Apigenin-C-hexoside-C-pentoside isomer 2 | n.d. | 0.126 ± 0.009 | n.d. | 4.058 ± 0.103 |
| Apigenin-C-hexoside-C-pentoside isomer 3 | n.d. | 0.030 ± 0.002 | n.d. | n.d. |
| Apigenin-*O*-rutinoside | 0.044 ± 0.003 | n.d. | n.d. | 5.115 ± 0.074 |
| Apigenin-*O*-hexoside-*O*-hexoside | 0.125 ± 0.004 | n.d. | n.d. | n.d. |
| Apigenin-C-hexoside-C-hexoside | n.d. | 0.135 ± 0.004 | 0.209 ± 0.025 | n.d. |
| Luteolin-7-*O*-rutinoside | 0.023 ± 0.001 | n.d. | n.d. | 411.706 ± 27.350 |
| Luteolin-*O*-hexoside-*O*-hexoside isomer 1 | 0.014 ± 0.001 | n.d. | n.d. | n.d. |
| Luteolin-*O*-hexoside-*O*-hexoside isomer 2 | 0.033 ± 0.001 | n.d. | n.d. | n.d. |
| Luteolin-*O*-glucuronide-*O*-hexoside isomer 1 | n.d. | n.d. | n.d. | 146.672 ± 2.333 |
| Luteolin-*O*-glucuronide-*O*-hexoside isomer 2 | n.d. | n.d. | n.d. | n.d. |
| Luteolin-*O*-glucuronide-*O*-hexoside isomer 3 | n.d. | n.d. | n.d. | n.d. |
| ***Total flavones*** | ***5.989 ± 0.244*** | ***4.928 ± 0.296*** | ***1.403 ± 0.159*** | ***3447.707 ± 97.914*** |

n.d. means that the compound was not detected in the sample.
