# Supplementary material for: Bioaccessibility of Flavones, Flavanones, and Flavonols from Vegetable Foods and Beverages
Source: Biology (Basel). 2024 Dec 22;13(12):1081. doi: 10.3390/biology13121081 (PMC11672976; doi:10.3390/biology13121081)
Supplement: Supplementary file 1 [file biology-13-01081-s001.zip › Supplementary Table S4.pdf]

Supplementary Table S4. Amount of flavonols in selected beverages and vegetable foods. Results are expressed in mg of flavonols/100g or 100 mL of vegetable foods or beverages.

Compound	Chamomile	Rooibos	Green Tea	Capers	Red-skinned Onion
Kaempferol	n.d.	n.d.	0.013 ± 0.000	n.d.	0.781 ± 0.011
Quercetin	0.022 ± 0.002	0.194 ± 0.015	0.024 ± 0.000	1.376 ± 0.091	n.d.
Methyl-quercetin isomer 1	0.091 ± 0.007	n.d.	n.d.	n.d.	n.d.
Methyl-quercetin isomer 2	0.068 ± 0.007	n.d.	n.d.	n.d.	n.d.
Methyl-quercetin isomer 3	0.041 ± 0.002	n.d.	n.d.	n.d.	n.d.
Methyl-quercetin isomer 4	0.009 ± 0.000	n.d.	n.d.	n.d.	n.d.
Isorhamnetin	0.014 ± 0.001	0.002 ± 0.000	n.d.	n.d.	0.104 ± 0.007
Myrcetin	n.d.	n.d.	0.009 ± 0.000	n.d.	n.d.
Kaempferol-3-O-rhamnoside	n.d.	n.d.	0.025 ± 0.001	n.d.	n.d.
Quercetin-3-O-pentoside	n.d.	n.d.	0.058 ± 0.005	0.019 ± 0.000	n.d.
Quercetin-3-O-rhamnoside isomer 1	n.d.	n.d.	n.d.	0.016 ± 0.001	n.d.
Quercetin-3-O-rhamnoside isomer 2	n.d.	n.d.	n.d.	0.010 ± 0.000	n.d.
Kaempferol-3-O-hexoside isomer 1	n.d.	n.d.	0.085 ± 0.015	n.d.	0.021 ± 0.001
Kaempferol-3-O-hexoside isomer 2	n.d.	n.d.	0.443 ± 0.015	n.d.	0.050 ± 0.001
Quercetin-O-hexoside isomer 1	0.067 ± 0.003	n.d.	n.d.	n.d.	n.d.
Quercetin-3-O-glucoside	0.779 ± 0.024	0.124 ± 0.008	1.143 ± 0.097	1.429 ± 0.048	1.538 ± 0.014
Quercetin-3-O-galactoside	n.d.	0.064 ± 0.001	n.d.	n.d.	n.d.
Quercetin-4'-O-glucoside	n.d.	n.d.	n.d.	n.d.	20.030 ± 0.915
Quercetin-O-hexoside isomer 2	n.d.	n.d.	n.d.	n.d.	n.d.
Quercetin-O-hexoside isomer 3	n.d.	n.d.	n.d.	n.d.	n.d.
Isorhamnetin-3-O-hexoside isomer 1	0.130 ± 0.010	n.d.	n.d.	n.d.	n.d.
Isorhamnetin-3-O-hexoside isomer 2	0.399 ± 0.023	n.d.	n.d.	n.d.	0.025 ± 0.001
Isorhamnetin-4'-O-hexoside	n.d.	n.d.	n.d.	n.d.	5.099 ± 0.084
Myricetin-3-O-hexoside	0.964 ± 0.038	n.d.	n.d.	0.007 ± 0.000	n.d.
Kaempferol-O-acetylhexoside isomer 1	0.005 ± 0.000	0.003 ± 0.000	n.d.	n.d.	n.d.
Kaempferol-O-acetylhexoside isomer 2	0.007 ± 0.000	0.005 ± 0.000	n.d.	n.d.	n.d.

Kaempferol-O-acetylhexoside isomer 3	0.008 ± 0.001	n.d.	n.d.	n.d.	n.d.
Kaempferol-O-acetylhexoside isomer 4	0.003 ± 0.000	n.d.	n.d.	n.d.	n.d.
Patuletin-3-O-hexoside	1.819 ± 0.049	n.d.	n.d.	n.d.	n.d.
Quercetin-O-acetylhexoside isomer 1	0.020 ± 0.001	n.d.	0.010 ± 0.000	n.d.	n.d.
Quercetin-O-acetylhexoside isomer 2	0.022 ± 0.002	n.d.	n.d.	n.d.	n.d.
Quercetin-O-acetylhexoside isomer 3	0.006 ± 0.000	n.d.	n.d.	n.d.	n.d.
Quercetin-3-O-malonyl-hexoside isomer 1	0.021 ± 0.001	n.d.	0.009 ± 0.000	n.d.	n.d.
Quercetin-3-O-malonyl-hexoside isomer 2	0.029 ± 0.002	n.d.	n.d.	n.d.	n.d.
Kaempferol-3-O-hexoside-rhmanoside	n.d.	n.d.	n.d.	1.965 ± 0.006	n.d.
Kaempferol-3-O-rutinoside	n.d.	n.d.	0.535 ± 0.040	132.867 ± 2.503	n.d.
Quercetin-O-hexoside-pentoside	n.d.	n.d.	n.d.	0.050 ± 0.001	n.d.
Kaempferol-O-hexoside-hexoside	n.d.	n.d.	n.d.	n.d.	0.063 ± 0.000
Quercetin-3-O-rutinoside	n.d.	1.011 ± 0.049	2.762 ± 0.192	150.057 ± 0.774	n.d.
Isorhamnetin-O-hexoside-O-pentoside	n.d.	n.d.	n.d.	n.d.	0.154 ± 0.005
Kaempferol-O-pentoside-O-acetylhexoside	n.d.	n.d.	n.d.	0.033 ± 0.001	n.d.
Isorhamnetin-3-O-rutinoside	n.d.	n.d.	n.d.	1.400 ± 0.018	n.d.
Quercetin-O-hexoside-hexoside isomer 1	n.d.	n.d.	n.d.	n.d.	n.d.
Quercetin-7-O-glucoside-4'-O-glucoside	n.d.	n.d.	n.d.	n.d.	0.371 ± 0.012
Quercetin-3-O-glucoside-4'-O-glucoside	n.d.	n.d.	n.d.	n.d.	13.753 ± 0.932
Quercetin-O-hexoside-hexoside isomer 2	n.d.	n.d.	n.d.	n.d.	n.d.
Quercetin-O-hexoside-hexoside isomer 3	n.d.	n.d.	n.d.	n.d.	n.d.

Quercetin-O-hexoside-hexoside isomer 4	n.d.	n.d.	n.d.	n.d.	n.d.
Quercetin-O-hexoside-hexoside isomer 5	0.008 ± 0.000	n.d.	n.d.	0.028 ± 0.001	n.d.
Quercetin-O-hexoside-hexoside isomer 6	n.d.	n.d.	n.d.	0.201 ± 0.009	n.d.
Kaempferol-O-rhamnoside-O-acetylhexoside isomer 1	n.d.	n.d.	n.d.	0.022 ± 0.001	n.d.
Kaempferol-O-rhamnoside-O-acetylhexoside isomer 2	n.d.	n.d.	n.d.	0.020 ± 0.002	n.d.
Isorhamnetin-3-O-hexoside-4'-O-hexoside	n.d.	n.d.	n.d.	n.d.	0.990 ± 0.017
Isorhamnetin-O-hexoside-O-hexoside isomer 1	n.d.	n.d.	n.d.	n.d.	n.d.
Isorhamnetin-O-hexoside-O-hexoside isomer 2	n.d.	n.d.	n.d.	n.d.	n.d.
Isorhamnetin-O-hexoside-O-hexoside isomer 3	n.d.	n.d.	n.d.	n.d.	n.d.
Myricetin-O-hexoside-O-hexoside	0.004 ± 0.000	n.d.	0.011 ± 0.001	n.d.	0.034 ± 0.001
Kaempferol-O-rhamnoside-O-rutinoside	n.d.	n.d.	0.039 ± 0.002	6.094 ± 0.061	n.d.
Quercetin-O-rutinoside-rhamnoside	n.d.	n.d.	n.d.	0.097 ± 0.002	n.d.
Kaempferol-O-hexoside-O-rutinoside isomer 1	n.d.	n.d.	n.d.	0.028 ± 0.002	n.d.
Kaempferol-O-hexoside-O-rutinoside isomer 2	n.d.	n.d.	n.d.	0.018 ± 0.001	n.d.
Kaempferol-O-hexoside-O-rutinoside isomer 3	n.d.	n.d.	0.135 ± 0.007	0.030 ± 0.002	n.d.
Kaempferol-O-hexoside-O-rutinoside isomer 4	n.d.	n.d.	0.191 ± 0.010	0.213 ± 0.013	n.d.
Quercetin-O-hexoside-O-rutinoside isomer 1	n.d.	n.d.	n.d.	0.056 ± 0.000	n.d.
Quercetin-O-hexoside-O-rutinoside isomer 2	n.d.	n.d.	0.237 ± 0.009	0.029 ± 0.002	n.d.

Quercetin-O-hexoside-O-rutinoside isomer 3	n.d.	n.d.	0.587 ± 0.033	0.183 ± 0.016	n.d.
Kaempferol-O-rhamnoside-O-rhamnoside-O-acetylhexoside	n.d.	n.d.	n.d.	0.025 ± 0.001	n.d.
Quercetin-tri-O-hexoside isomer 1	n.d.	n.d.	n.d.	n.d.	n.d.
Quercetin-tri-O-hexoside isomer 2	n.d.	n.d.	n.d.	n.d.	n.d.
Quercetin-tri-O-hexoside isomer 3	n.d.	n.d.	n.d.	n.d.	n.d.
Quercetin-tri-O-hexoside isomer 4	n.d.	n.d.	n.d.	n.d.	n.d.
Quercetin-tri-O-hexoside isomer 5	n.d.	n.d.	n.d.	n.d.	n.d.
Quercetin-tri-O-hexoside isomer 6	n.d.	n.d.	n.d.	n.d.	0.114 ± 0.001
Total flavonols	***4.536 ± 0.176***	***1.403 ± 0.073***	***6.316 ± 0.429***	***296.272 ± 3.558***	***43.144 ± 2.003***

n.d. means that the compound was not detected in the sample.
