# Supplementary material for: Bioaccessibility of Flavones, Flavanones, and Flavonols from Vegetable Foods and Beverages
Source: Biology (Basel). 2024 Dec 22;13(12):1081. doi: 10.3390/biology13121081 (PMC11672976; doi:10.3390/biology13121081)
Supplement: Supplementary file 1 [file biology-13-01081-s001.zip › Supplementary Table S5.pdf]

**Supplementary Table S5.** Amount of flavanones in selected beverages and vegetable foods after *in vitro* gastro-intestinal digestion. Results are expressed in mg of flavanones/100g or 100 mL of vegetable foods or beverages. Bioaccessibility index (BI) is the percentage ratio between the concentration after *in vitro* gastro-intestinal digestion and the concentration in the methanolic extract.

| **Compound** | ***Blonde Orange*** | | ***Blood Orange*** | | ***Orange Juice*** | | ***Rooibos*** | |
|---|---|---|---|---|---|---|---|---|
| | | **BI (%)** | | BI (%) | | BI (%) | | BI (%) |
| Tri-hydroxy-flavanone | 0.021 ± 0.000 | n.f. | 0.008 ± 0.002 | n.f. | 0.006 ± 0.001 | n.f. | n.d | n.d |
| Naringenin | 0.017 ± 0.001 | n.f. | 0.009 ± 0.001 | n.f. | 0.001 ± 0.000 | n.f. | 0.003 ± 0.000* | 46.18 |
| Tetra-hydroxy-flavanone | n.d | n.d | n.d | n.d | n.d | n.d | 0.002 ± 0.000* | 6.41 |
| Naringenin-C-hexoside isomer 1 | n.d | n.d | n.d | n.d | n.d | n.d | n.d | 0.00 |
| Naringenin-C-hexoside isomer 2 | n.d | n.d | n.d | n.d | n.d | n.d | n.d | 0.00 |
| Naringenin-C-hexoside isomer 3 | n.d | n.d | n.d | n.d | n.d | n.d | n.d | 0.00 |
| Naringenin-*O*-hexoside | 0.172 ± 0.015* | 80.34. | 0.056 ± 0.005* | 200.43 | 0.052 ± 0.002 | 84.46 | n.d | n.d. |
| Tetra-hydroxyflavanone-C-hexoside isomer 1 | n.d | n.d | n.d | n.d | n.d | n.d | 0.077 ± 0.003* | 10.96. |
| Tetra-hydroxyflavanone-C-hexoside isomer 2 | n.d | n.d | n.d | n.d | n.d | n.d | 0.037 ± 0.002* | 4.96 |
| Tetra-hydroxyflavanone-C-hexoside isomer 3 | n.d | n.d | n.d | n.d | n.d | n.d | 0.012 ± 0.001* | 3.18 |
| Tetra-hydroxyflavanone-C-hexoside isomer 4 | n.d | n.d | n.d | n.d | n.d | n.d | 0.009 ± 0.001* | 5.83 |
| Tetra-hydroxyflavanone-*O*-hexoside isomer 1 | 0.013 ± 0.001* | 68.27 | 0.016 ± 0.001 | 112.23 | 0.008 ± 0.001 | 89.14 | n.d. | n.d. |
| Tetra-hydroxyflavanone-*O*-hexoside isomer 2 | 0.011 ± 0.005 | 92.47 | 0.010 ± 0.000 | 75.55 | 0.002 ± 0.000 | n.f. | n.d. | n.d. |
| Penta-hydroxyflavanone-*O*-hexoside isomer 1 | n.d | n.d | 0.098 ± 0.001* | 327.39 | 0.001 ± 0.001 | n.f. | n.d. | n.d. |
| Penta-hydroxyflavanone-*O*-hexoside isomer 2 | n.d | n.d | 0.019 ± 0.002* | 39.36 | 0.001 ± 0.000 | n.f. | n.d. | n.d. |
| Naringenin-7-*O*-neohesperidoside | 12.585 ± 0.294 | 105.14 | 5.050 ± 0.040* | 77.53 | 4.016 ± 0.467* | 133.62 | n.d. | n.d. |
| Naringenin-di-C-hexoside | n.d | n.d | n.d | n.d | n.d | n.d | n.d. | 0.00 |

| | | | | | | | | |
|---|---|---|---|---|---|---|---|---|
| Naringenin-*O*-hexoside-*O*-hexoside | 0.240 ± 0.019* | 80.72 | 0.189 ± 0.005 | 108.07 | 0.238 ± 0.030 | 108.28 | n.d. | n.d. |
| Hesperetin-7-*O*-rutinoside | 32.061 ± 2.054 | 94.73 | 24.154 ± 2.336* | 77.53 | 9.941 ± 0.518* | 165.57 | n.d. | n.d. |
| Tetra-hydroxyflavanone-di-C-hexoside | n.d | n.d | n.d | n.d | n.d. | n.d. | 0.011 ± 0.001* | 7.63 |
| Narigenin-*O*-acetylhexoside-*O*-pentoside-pentoside | 0.118 ± 0.025 | 110.62 | 0.058 ± 0.002* | 150.96 | 0.029 ± 0.002 | 105.80 | n.d. | n.d. |
| Naringenin-*O*-hexoside-*O*-rutinoside | 0.404 ± 0.042 | 89.91 | 0.125 ± 0.010* | 67.92 | 0.144 ± 0.004 | 105.32 | n.d. | n.d. |
| Hesperetin-*O*-rutinoside-*O*-hexoside | 0.126 ± 0.014* | 74.37 | 0.092 ± 0.005* | 72.27 | 0.170 ± 0.007 | 107.27 | n.d. | n.d. |
| ***Total flavanones*** | ***45.766 ± 2.470*** | ***97.21*** | ***29.885 ± 2.407**** | ***78.20*** | ***14.609 ± 1.052**** | ***151.72*** | ***0.151 ± 0.007**** | ***6.79*** |

Asterisk indicated significant differences (*P<0.05*) between the same compound before digestion.
n.d. means that the compound was not detected in the sample; n.f. means newly formed compound.
