# Supplementary material for: Bioaccessibility of Flavones, Flavanones, and Flavonols from Vegetable Foods and Beverages
Source: Biology (Basel). 2024 Dec 22;13(12):1081. doi: 10.3390/biology13121081 (PMC11672976; doi:10.3390/biology13121081)
Supplement: Supplementary file 1 [file biology-13-01081-s001.zip › Supplementary Table S6.pdf]

Supplementary Table S6. Amount of flavones in selected beverages and vegetable foods after *in vitro* gastro-intestinal digestion. Results are expressed in mg of flavones/100g or 100 mL of vegetable foods or beverages. Bioaccessibility index (BI) is the percentage ratio between the concentration after *in vitro* gastro-intestinal digestion and the concentration in the methanolic extract.

Compound	Chamomile		Rooibos		Green Tea		Red Radicchio	
		BI (%)		BI (%)		BI (%)		BI (%)
Apigenin	0.993 ± 0.082*	140.05	0.008 ± 0.001*	47.30	n.d.	n.d.	n.d.	n.d
Luteolin	0.010 ± 0.001	76.18	0.083 ± 0.010*	68.62	n.d.	n.d.	38.380 ± 2.232	47975
Tri-hydroxy-methoxyflavone isomer 1	0.045 ± 0.005	116.50	n.d	0.00	n.d.	n.d	n.d.	n.d.
Tri-hydroxy-methoxyflavone isomer 2	0.06 ± 0.001*	56.15	n.d.	n.d	n.d.	n.d	n.d.	n.d.
Tri-hydroxy-methoxyflavone isomer 3	0.009 ± 0.002	130.42	n.d.	n.d	n.d.	n.d.	n.d.	n.d.
Tri-hydroxy-methoxyflavone isomer 4	0.014 ± 0.002	130.85	0.050 ± 0.009	95.77	n.d.	n.d	n.d.	n.d.
Apigenin-C-hexoside	n.d.	n.d	0.820 ± 0.049*	80.45	0.250 ± 0.021*	79.66	9.908 ± 0.069*	63.07
Apigenin-7-*O*-glucoside	3.549 ± 0.283*	165.85	n.d.	n.d	n.d.	n.d.	3.493 ± 0.618*	45.50
Apigenin-*O*-glucuronide	n.d.	n.d.	n.d.	n.d	n.d.	n.d.	22.382 ± 0.337*	31.53
Luteolin-7-*O*-glucoside	0.473 ± 0.009*	70.84	n.d.	n.d	n.d.	n.d.	1106.613 ± 13.310*	78.65
Luteolin-*O*-hexoside isomer 1	n.d.	n.d	n.d.	n.d	n.d.	n.d.	62.208 ± 1.658*	250.62
Luteolin-*O*-hexoside isomer 2	n.d.	n.d.	n.d.	n.d	n.d.	n.d.	117.932 ± 1.696*	308.79
Luteolin-*O*-hexoside isomer 3	n.d.	n.d	n.d.	n.d	n.d.	n.d.	37.179 ± 0.777	n.f.
Luteolin-C-hexoside isomer 1	n.d.	n.d.	0.615 ± 0.054*	35.21	n.d.	0.00	n.d.	n.d.
Luteolin-C-hexoside isomer 2	n.d.	n.d.	1.598 ± 0.026	96.09	n.d.	0.00	n.d.	n.d.
Luteolin-C-hexoside isomer 3	n.d.	n.d	n.d.	n.d	n.d.	n.d.	n.d.	0.00
Luteolin-7-*O*-glucuronide	n.d.	n.d.	n.d.	n.d	n.d.	n.d.	1018.663 ± 9.776*	77.61
Luteolin-*O*-glucuronide isomer 1	n.d.	n.d.	n.d.	n.d	n.d.	n.d.	11.391 ± 0.163	n.f.
Luteolin-*O*-glucuronide isomer 2	n.d.	n.d.	n.d.	n.d	n.d.	n.d.	283.339 ± 8.713	n.f.

Luteolin-*O*-glucuronide isomer 3	n.d.	n.d.	n.d.	n.d	n.d.	n.d.	200.052 ± 8.009	n.f.
Apigenin-*O*-acetylhexoside isomer 1	n.d.	0.00	n.d.	n.d	n.d.	n.d.	n.d.	n.d.
Apigenin-*O*-acetylhexoside isomer 2	0.394 ± 0.007*	132.35	n.d.	n.d	n.d.	n.d.	n.d.	n.d.
Apigenin-*O*-acetylhexoside isomer 3	0.809 ± 0.054	111.61	n.d.	n.d	n.d.	n.d.	n.d.	n.d.
Apigenin-*O*-diacetylhexoside	0.453 ± 0.040*	55.47	n.d.	n.d	n.d.	n.d.	n.d.	n.d.
Apigenin-*O*-malonylhexoside	n.d.	0.00	n.d.	n.d	n.d.	n.d.	n.d.	n.d.
Luteolin-*O*-malonylhexoside	0.043 ± 0.005	111.53	0.101 ± 0.009*	757.98	n.d.	n.d.	n.d.	n.d.
Apigenin-C-hexoside-C-pentoside isomer 1	0.016 ± 0.001	93.37	n.d.	n.d	0.612 ± 0.056*	74.68	n.d.	n.d.
Apigenin-C-hexoside-C-pentoside isomer 2	n.d.	n.d.	0.105 ± 0.028	83.30	n.d.	n.d.	3.388 ± 0.265*	83.50
Apigenin-C-hexoside-C-pentoside isomer 3	n.d.	n.d.	0.180 ± 0.022*	598.587	n.d.	n.d.	n.d.	n.d.
Apigenin-*O*-rutinoside	0.050 ± 0.008	112.70	n.d.	n.d	n.d.	n.d.	2.933 ± 0.351*	57.34
Apigenin-*O*-hexoside-*O*-hexoside	0.084 ± 0.012*	66.95	n.d.	n.d	n.d.	n.d.	n.d.	n.d.
Apigenin-C-hexoside-C-hexoside	n.d.	n.d.	0.122 ± 0.004*	90.23	0.145 ± 0.014*	69.39	n.d.	n.d.
Luteolin-7-*O*-rutinoside	n.d.	0.00	n.d.	n.d	n.d.	n.d.	396.230 ± 3.062	96.24
Luteolin-*O*-hexoside-*O*-hexoside isomer 1	n.d.	0.00	n.d.	n.d	n.d.	n.d.	n.d.	n.d.
Luteolin-*O*-hexoside-*O*-hexoside isomer 2	n.d.	0.00	n.d.	n.d	n.d.	n.d.	25.152 ± 0.824	n.f.
Luteolin-*O*-glucuronide-*O*-hexoside isomer 1	n.d.	n.d.	n.d.	n.d	n.d.	n.d.	145.738 ± 1.233	99.36
Luteolin-*O*-glucuronide-*O*-hexoside isomer 2	n.d.	n.d.	n.d.	n.d	n.d.	n.d.	31.246 ± 1.881	n.f.
Luteolin-*O*-glucuronide-*O*-	n.d.	n.d.	n.d.	n.d	n.d.	n.d.	13.298 ± 0.150	n.f.

hexoside isomer 3							
Total flavones	***6.946 ± 0.511***	***115.98***	***3.683 ± 0.212****	***74.73***	***1.007 ± 0.091****	***71.79***	***3529.525 ± 54.724***	***102.37***

Asterisk indicated significant differences (*P<0.05*) between the same compound before digestion.

n.d. means that the compound was not detected in the sample; n.f. means newly formed compound
