# Supplementary material for: Bioaccessibility of Flavones, Flavanones, and Flavonols from Vegetable Foods and Beverages
Source: Biology (Basel). 2024 Dec 22;13(12):1081. doi: 10.3390/biology13121081 (PMC11672976; doi:10.3390/biology13121081)
Supplement: Supplementary file 1 [file biology-13-01081-s001.zip › Supplementary Table S7.pdf]

Supplementary Table S7. Amount of flavonols in selected beverages and vegetable foods after *in vitro* gastro-intestinal digestion. Results are expressed in mg of flavonols/100g or 100 mL of vegetable foods or beverages. Bioaccessibility index (BI) is the percentage ratio between the concentration after *in vitro* gastro-intestinal digestion and the concentration in the methanolic extract.

Compound	Chamomile		Rooibos		Green Tea		Capers		Red-skinned Onion	
		BI (%)		BI (%)		BI (%)		BI (%)		BI (%)
Kaempferol	n.d.	n.d	n.d.	n.d	n.d.	0.00	n.d.	n.d	n.d.	n.d.
Quercetin	n.d.	0.00	n.d.	0.00	n.d.	0.00	n.d.	0.00	2.135 ± 0.021*	273.32
Methyl-quercetin isomer 1	n.d.	0.00	n.d.	n.d	n.d.	n.d	n.d.	n.d.	n.d.	n.d.
Methyl-quercetin isomer 2	n.d.	0.00	n.d.	n.d	n.d.	n.d	n.d.	n.d.	n.d.	n.d.
Methyl-quercetin isomer 3	n.d.	0.00	n.d.	n.d	n.d.	n.d	n.d.	n.d.	n.d.	n.d.
Methyl-quercetin isomer 4	n.d.	0.00	n.d.	n.d	n.d	n.d	n.d.	n.d.	n.d.	n.d.
Isorhamnetin	n.d.	0.00	n.d.	0.00	n.d.	n.d	n.d.	n.d.	0.011 ± 0.000*	10.91
Myrcetin	n.d.	n.d	n.d.	n.d	n.d.	0.00	n.d.	n.d.	n.d.	n.d.
Kaempferol-3-O-rhamnoside	n.d.	n.d	n.d.	n.d	n.d.	0.00	n.d.	n.d.	n.d.	n.d.
Quercetin-3-O-pentoside	n.d.	n.d	n.d.	n.d	n.d.	0.00	n.d.	0.00	n.d.	n.d.
Quercetin-3-O-rhamnoside isomer 1	n.d.	n.d	n.d.	n.d	n.d.	n.d.	n.d.	0.00	n.d.	n.d.
Quercetin-3-O-rhamnoside isomer 2	n.d.	n.d	n.d.	n.d	n.d.	n.d.	n.d.	0.00	n.d.	n.d.
Kaempferol-3-O-hexoside isomer 1	n.d.	n.d	n.d.	n.d	n.d.	0.00	n.d.	n.d.	n.d.	0.00
Kaempferol-3-O-hexoside isomer 2	n.d.	n.d	n.d.	n.d	0.294 ± 0.010*	66.38	n.d.	n.d.	0.015 ± 0.001*	29.50
Quercetin-O-hexoside isomer 1	n.d.	0.00	n.d.	n.d	n.d.	n.d.	n.d.	n.d.	n.d.	n.d.
Quercetin-3-O-glucoside	0.067 ± 0.011*	8.57	0.018 ± 0.005*	14.38	0.031 ± 0.003*	2.75	0.053 ± 0.005*	3.68	5.319 ± 0.287*	345.84
Quercetin-3-O-galactoside	n.d.	n.d.	0.017 ± 0.006	26.41	n.d.	n.d.	n.d.	n.d.	n.d.	n.d.
Quercetin-4'-O-glucoside	n.d.	n.d.	n.d.	n.d.	n.d.	n.d.	n.d.	n.d.	20.705 ± 0.342	103.37

Quercetin-O-hexoside isomer 2	n.d.	n.d	n.d.	n.d	n.d.	n.d.	n.d.	n.d.	0.050 ± 0.005	n.f.
Quercetin-O-hexoside isomer 3	n.d.	n.d.	n.d.	n.d	n.d.	n.d.	n.d.	n.d.	0.037 ± 0.000	n.f.
Isorhamnetin-3-O-hexoside isomer 1	0.018 ± 0.004*	13.47	n.d.	n.d	n.d.	n.d.	n.d.	n.d.	n.d.	n.d.
Isorhamnetin-3-*O*-hexoside isomer 2	0.118 ± 0.014*	29.64	n.d.	n.d	n.d.	n.d.	n.d.	n.d.	0.203 ± 0.004*	799.98
Isorhamnetin-4'-O-hexoside	n.d.	n.d.	n.d.	n.d	n.d.	n.d.	n.d.	n.d.	6.939 ± 0.111*	136.08
Myricetin-3-O-hexoside	n.d.	0.00	n.d.	n.d	n.d.	n.d.	n.d.	0.00	n.d.	n.d.
Kaempferol-O-acetylhexoside isomer 1	n.d.	0.00	n.d.	0.00	n.d.	n.d.	n.d.	n.d.	n.d.	n.d.
Kaempferol-O-acetylhexoside isomer 2	n.d.	0.00	n.d.	0.00	n.d.	n.d.	n.d.	n.d.	n.d.	n.d.
Kaempferol-O-acetylhexoside isomer 3	n.d.	0.00	n.d.	n.d	n.d.	n.d.	n.d.	n.d.	n.d.	n.d.
Kaempferol-O-acetylhexoside isomer 4	n.d.	0.00	n.d.	n.d	n.d.	n.d.	n.d.	n.d.	n.d.	n.d.
Patuletin-3-O-hexoside	0.018 ± 0.001*	1.01	n.d.	n.d	n.d.	n.d.	n.d.	n.d.	n.d.	n.d.
Quercetin-O-acetylhexoside isomer 1	n.d.	0.00	n.d.	n.d	n.d.	0.00	n.d.	n.d.	n.d.	n.d.
Quercetin-O-acetylhexoside isomer 2	0.006 ± 0.001*	27.60	n.d.	n.d	n.d.	n.d.	n.d.	n.d.	n.d.	n.d.
Quercetin-O-acetylhexoside isomer 3	n.d.	0.00	n.d.	n.d	n.d.	n.d.	n.d.	n.d.	n.d.	n.d.
Quercetin-3-O-malonyl-hexoside isomer 1	n.d.	0.00	n.d.	n.d	n.d.	0.00	n.d.	n.d.	n.d.	n.d.
Quercetin-3-O-malonyl-hexoside isomer 2	0.009 ± 0.000*	31.88	n.d.	n.d	n.d.	n.d.	n.d.	n.d.	n.d.	n.d.

Kaempferol-3-O-hexoside-rhmanoside	n.d.	n.d.	n.d.	n.d	n.d.	n.d.	0.372 ± 0.006*	18.94	n.d.	n.d.
Kaempferol-3-O-rutinoside	n.d.	n.d.	n.d.	n.d	0.373 ± 0.033*	69.66	120.927 ± 4.740*	91.01	n.d.	n.d.
Quercetin-O-hexoside-pentoside	n.d.	n.d.	n.d.	n.d	n.d.	n.d.	n.d.	0.00	n.d.	n.d.
Kaempferol-O-hexoside-hexoside	n.d.	n.d.	n.d.	n.d	n.d.	n.d.	n.d.	n.d.	0.011 ± 0.000*	17.18
Quercetin-3-O-rutinoside	n.d.	n.d.	0.652 ± 0.079*	64.51	2.027 ± 0.095*	72.93	71.680 ± 6.077*	47.77	n.d.	0.00
Isorhamnetin-O-hexoside-O-pentoside	n.d.	n.d.	n.d.	n.d	n.d.	n.d.	n.d.	n.d.	0.010 ± 0.001*	6.37
Kaempferol-O-pentoside-O-acetylhexoside	n.d.	n.d.	n.d.	n.d	n.d.	n.d.	n.d.	0.00	n.d.	n.d.
Isorhamnetin-3-O-rutinoside	n.d.	n.d.	n.d.	n.d	n.d	n.d.	0.328 ± 0.002*	23.41	n.d.	n.d.
Quercetin-O-hexoside-hexoside isomer 1	n.d.	n.d.	n.d.	n.d	n.d	n.d.	n.d	n.d.	0.034 ± 0.001*	n.f.
Quercetin-7-O-glucoside-4'-O-glucoside	n.d.	n.d.	n.d.	n.d	n.d	n.d.	n.d	n.d.	0.099 ± 0.004*	26.56
Quercetin-3-O-glucoside-4'-O-glucoside	n.d.	n.d.	n.d.	n.d	n.d	n.d.	n.d	n.d.	17.874 ± 0.542*	129.97
Quercetin-O-hexoside-hexoside isomer 2	n.d.	n.d.	n.d.	n.d	n.d	n.d.	n.d	n.d.	0.096 ± 0.004	n.f.
Quercetin-O-hexoside-hexoside isomer 3	n.d.	n.d.	n.d.	n.d	n.d	n.d.	n.d	n.d.	0.012 ± 0.001	n.f.
Quercetin-O-hexoside-hexoside isomer 4	n.d.	n.d.	n.d.	n.d	n.d	n.d.	n.d	n.d.	0.106 ± 0.006	n.f.
Quercetin-O-hexoside-hexoside isomer 5	n.d.	0.00	n.d.	n.d	n.d	n.d.	n.d	0.00	0.025 ± 0.001	n.f.
Quercetin-O-hexoside-	n.d.	n.d.	n.d.	n.d	n.d	n.d.	0.021 ± 0.001*	10.39	0.109 ± 0.009	n.f.

hexoside isomer 6										
Kaempferol-O-rhamnoside-O-acetylhexoside isomer 1	n.d.	n.d.	n.d.	n.d	n.d	n.d.	n.d	0.00	n.d	n.d.
Kaempferol-O-rhamnoside-O-acetylhexoside isomer 2	n.d.	n.d.	n.d.	n.d	n.d	n.d.	n.d	0.00	n.d	n.d.
Isorhamnetin-3-O-hexoside-4'-O-hexoside	n.d.	n.d.	n.d.	n.d	n.d	n.d.	n.d	n.d.	0.420 ± 0.005*	42.48
Isorhamnetin-O-hexoside-O-hexoside isomer 1	n.d.	n.d.	n.d.	n.d	n.d	n.d.	n.d	n.d.	0.011 ± 0.001	n.f.
Isorhamnetin-O-hexoside-O-hexoside isomer 2	n.d.	n.d.	n.d.	n.d	n.d	n.d.	n.d	n.d.	0.016 ± 0.001	n.f.
Isorhamnetin-O-hexoside-O-hexoside isomer 3	n.d.	n.d.	n.d.	n.d	n.d	n.d.	n.d	n.d.	0.174 ± 0.001	n.f.
Myricetin-O-hexoside-O-hexoside	n.d.	0.00	n.d.	n.d	n.d.	0.00	n.d	n.d.	n.d.	0.00
Kaempferol-O-rhamnoside-O-rutinoside	n.d.	n.d.	n.d.	n.d	n.d.	0.00	6.021 ± 0.423	98.80	n.d	n.d.
Quercetin-O-rutinoside-rhamnoside	n.d.	n.d.	n.d.	n.d	n.d	n.d.	0.120 ± 0.006*	123.09	n.d	n.d.
Kaempferol-O-hexoside-O-rutinoside isomer 1	n.d.	n.d.	n.d.	n.d	n.d	n.d.	0.028 ± 0.001	101.71	n.d	n.d.
Kaempferol-O-hexoside-O-rutinoside isomer 2	n.d.	n.d.	n.d.	n.d	n.d	n.d.	n.d.	0.00	n.d	n.d.
Kaempferol-O-hexoside-O-rutinoside isomer 3	n.d.	n.d.	n.d.	n.d	0.071 ± 0.004*	52.25	0.173 ± 0.003*	581.10	n.d	n.d.
Kaempferol-O-hexoside-	n.d.	n.d.	n.d.	n.d	0.047 ± 0.003*	24.89	4.230 ± 0.055*	1990.5	n.d	n.d.

O-rutinoside isomer 4										
Quercetin-O-hexoside-O-rutinoside isomer 1	n.d.	n.d.	n.d.	n.d	n.d.	n.d	0.098 ± 0.005*	176.02	n.d	n.d.
Quercetin-O-hexoside-O-rutinoside isomer 2	n.d.	n.d.	n.d.	n.d	n.d.	0.00	0.185 ± 0.010*	640.42	n.d	n.d.
Quercetin-O-hexoside-O-rutinoside isomer 3	n.d.	n.d.	n.d.	n.d	n.d.	0.00	2.336 ± 0.056*	1276.4	n.d	n.d.
Kaempferol-O-rhamnoside-O-rhamnoside-O-acetylhexoside	n.d.	n.d.	n.d.	n.d	n.d.	n.d	0.023 ± 0.000	93.22	n.d	n.d.
Quercetin-tri-O-hexoside isomer 1	n.d.	n.d.	n.d.	n.d	n.d.	n.d	n.d.	n.d	0.072 ± 0.005	n.f.
Quercetin-tri-O-hexoside isomer 2	n.d.	n.d.	n.d.	n.d	n.d.	n.d	n.d.	n.d	0.019 ± 0.001	n.f.
Quercetin-tri-O-hexoside isomer 3	n.d.	n.d.	n.d.	n.d	n.d.	n.d	n.d.	n.d	0.022 ± 0.000	n.f.
Quercetin-tri-O-hexoside isomer 4	n.d.	n.d.	n.d.	n.d	n.d.	n.d	n.d.	n.d	0.262 ± 0.001	n.f.
Quercetin-tri-O-hexoside isomer 5	n.d.	n.d.	n.d.	n.d	n.d.	n.d	n.d.	n.d	0.438 ± 0.004	n.f.
Quercetin-tri-O-hexoside isomer 6	n.d.	n.d.	n.d.	n.d	n.d.	n.d	n.d.	n.d	0.438 ± 0.013*	383.10
Total flavonols	***0.236 ± 0.032****	***5.21***	***0.687 ± 0.090****	***48.96***	***2.843 ± 0.059****	***44.81***	***206.594 ± 11.391****	***69.73***	***55.657 ± 1.370****	***129.00***

Asterisk indicated significant differences (*P<0.05*) between the same compound before digestion.
n.d. means that the compound was not detected in the sample; n.f. means newly formed compound.
